# Augmented Renal Clearance and Hypoalbuminemia-Induced Low Vancomycin Trough Concentrations in Febrile Neutropenic Patients With Hematological Malignancies

**DOI:** 10.7759/cureus.29568

**Published:** 2022-09-25

**Authors:** Abdullah M Alzahrani, Alqassem Y Hakami, Aeshah AlAzmi, Shahid Karim, Ahmed S Ali, Abdulhadi S Burzangi, Huda M Alkreathy, Mansoor A Khan, Rami M Alzhrani, Samah S Basudan, Yahya A Alzahrani

**Affiliations:** 1 Pharmaceutical Care Department, Ministry of National Guard - Health Affairs, Jeddah, SAU; 2 Department of Pharmacology, Faculty of Medicine, King Abdulaziz University, Jeddah, SAU; 3 College of Medicine, King Saud bin Abdulaziz University for Health Sciences, Jeddah, SAU; 4 Research Office, King Abdullah International Medical Research Center, Jeddah, SAU; 5 Department of Pharmaceutics and Industrial Pharmacy, College of Pharmacy, Taif University, Taif, SAU; 6 Department of Pharmacy, King Abdullah Medical Complex, Ministry of Health, Jeddah, SAU; 7 Drug Information Center, East Jeddah Hospital, Ministry of Health, Jeddah, SAU

**Keywords:** hypoalbuminemia, hematological malignancy, vancomycin low trough, febrile neutropenia, augmented renal clearance

## Abstract

Introduction: Vancomycin administration in individuals with hematological malignancy or neutropenia is associated with a suboptimal trough concentration. Nonetheless, most studies did not distinguish whether low vancomycin trough concentrations were due to hematological malignancies or neutropenia. This study aimed to determine the association between types of hematological malignancy and febrile neutropenia with low vancomycin concentrations.

Methods: The present retrospective chart review study was conducted by using clinical data adopted from computerized physician order entries (BestCare®) for all of the patients who received intravenous vancomycin treatment between January 2017 and December 2020 at King Abdulaziz Medical City in Jeddah.

Results: Out of the 296 patients, 217 were included. There was no significant association between the type of hematological malignancy and the incidence of a low trough concentration (p > 0.05), while a significant association between febrile neutropenia and the incidence of a low trough concentration was observed (p < 0.05). Furthermore, the predictors for a low trough among febrile neutropenic patients were creatinine clearance (CrCI) and a low albumin concentration. In addition, there was a significant association between febrile neutropenia and augmented renal clearance (p < 0.05).

Conclusions: The findings of this study conclude that febrile neutropenia is associated with low vancomycin concentrations. Interestingly, augmented renal clearance was observed in most of the febrile neutropenia patients with a significant association, which is considered the main driver for a low trough in neutropenic patients.

## Introduction

In recent decades, major advances have been achieved in treating patients with neoplastic diseases. Several chemotherapeutic agents and treatment modalities are available to obtain maximum antitumor responses. These agents can cause serious side effects, such as immunosuppression, which makes a patient highly vulnerable to infection [1]. Patients diagnosed with hematological malignancy (HM) frequently present with neutropenia symptoms, which are attributed to chemotherapy use [2]. Febrile neutropenia (FN) is a medical emergency complication of chemotherapy that can delay cancer treatment and, in some cases, reduce chemotherapy doses, which might lead to a decrease in treatment efficacy [3]. The immediate initiation of empirical antibiotic therapy is strongly recommended for febrile neutropenic patients. Vancomycin is a preferred choice for certain indications, such as hemodynamic instability, pneumonia, and catheter-associated infection. The initiation of antimicrobial therapy in a proper dose is crucial and influences a patient’s outcome to a large extent [4].

Recent investigations have shown that critically ill, septic [5], febrile neutropenic [6], and hematologic malignant patients [7] have an increase in renal clearance, a condition called augmented renal clearance (ARC). ARC is a pathological condition associated with enhancing renally excreted drugs’ filtration, such as that of antipseudomonal beta-lactams, aminoglycosides, and vancomycin [8]. Thus, these critical drugs will be administered at a subtherapeutic concentration, which may enhance the risk of treatment failure [9,10]. The evidence shows that a traditional vancomycin dose in individuals with an HM or FN is associated with a suboptimal trough concentration. Nonetheless, most of the studies did not distinguish whether the low vancomycin trough concentration was due to an HM or FN [11]. Furthermore, several studies have been conducted to determine the risk factors for ARC among critically ill patients, burn patients, and patients with neurotrauma [9,12,13]. No study has determined the risk factors for ARC among HM patients without mixed conditions. In light of this, understanding the actual causes of such phenomena would enable the identification of patients at a high risk of having subtherapeutic trough concentrations while receiving a conventional dose, e.g., those requiring a higher dose to receive adequate systemic exposure, particularly patients with a confirmed infection.

The Princess Norah Oncology Center (PNOC) is the leading and largest cancer center in the western region of Saudi Arabia, serving the western, northern, and southern regions of the Kingdom. However, over the last 20 years since the PNOC was inaugurated, no study has been conducted to evaluate the association between a low vancomycin trough concentration and an HM with or without FN. Moreover, with few exceptions, little has been undertaken by other centers around the Kingdom to address and evaluate this problem. Therefore, this study aimed to determine the association between types of HM, and FN with low vancomycin concentrations. Additionally, the risk factors for ARC among HM patients have been identified.

## Materials and methods

Study design and subjects

This study was approved by the Ethics Committee of the King Abdullah International Medical Research Center (KAIMRC) with the approval number SP21J/202/05. The present work was designed as a retrospective chart review, conducted using clinical data adopted from computerized physician order entries (BestCare®) for all patients who received intravenous vancomycin treatment between January 1, 2017, and December 31, 2020, at the King Abdulaziz Medical City at Jeddah (KAMC-J).

All adult HM patients with or without FN were included in this study, while patients were excluded if they were children, admitted to the ICU, required renal replacement therapy/hemodialysis, or had missing medical data. The patients’ demographics were age, sex, height, weight, serum creatinine concentration (µmol/L), BMI, type of therapy (treatment or empirical), type of HM, WBCs, neutrophile account, albumin concentration (g/dL), and steady-state trough concentration (μg/mL). Creatinine clearance (CrCI) was calculated using the Cockcroft-Gault equation where CrCl ={((140-age) x weight)/(72xSCr)}x 0.85 (if female) [14]. Vancomycin blood sampling was performed at a steady state at least 30 min before the fourth dose [15,16].

Study variables

The hematological malignancies were classified into leukemia, lymphoma, and multiple myeloma. FN is defined as “a one-time oral temperature of greater than 38.3°C (approximately 100.9°F) or a sustained temperature of greater than 38°C (100.4°F) for > 1 hour in a patient who has an absolute neutrophil count of less than 500 cells/mL, or an absolute neutrophil count expected to decrease to less than 500 cells/mL within 48 hours.” [17,18]. The vancomycin trough concentration was classified into three categories: low trough, < 10 μg/mL; normal trough, 10-20 μg/mL; and high trough, > 20 μg/mL. ARC is defined as a CrCl greater than the normal values of 130 ml/min in males and 120 ml/min in females [9].

Study endpoints

The primary endpoint was association of the type of HMs and FN with low vancomycin trough concentration.

Secondary endpoints were possible underlying factors of a low vancomycin trough concentration among patients with FN and the effect of ARC on the vancomycin trough concentration among FN patients.

Statistical analysis

The Statistical Package for the Social Sciences (SPSS) version 26.0 (SPSS Inc, Armonk, NY, USA) was used for the statistical analysis in this study. All continuous data were tested for normality using a histogram and the Shapiro-Wilk test. Demographic data were expressed as frequencies and percentages for the categorical variables, and the continuous variables were presented as the mean ± SE or median (interquartile range) where applicable. The student’s t-test was used to compare normally distributed groups, while the Mann-Whitney U and Kruskal-Wallis tests were used for those that were not normally distributed. The association between the two groups was performed using a contingency table analysis with a χ2 test. The odds ratios and adjusted odds ratio with a 95% confidence interval (95% CI) of the low trough concentration were calculated wherever appropriate. A multivariate logistic regression analysis was performed to determine the independence of each predictor of a low trough concentration. A p-value of < 0.05 was considered statistically significant.

## Results

**Figure 1:**
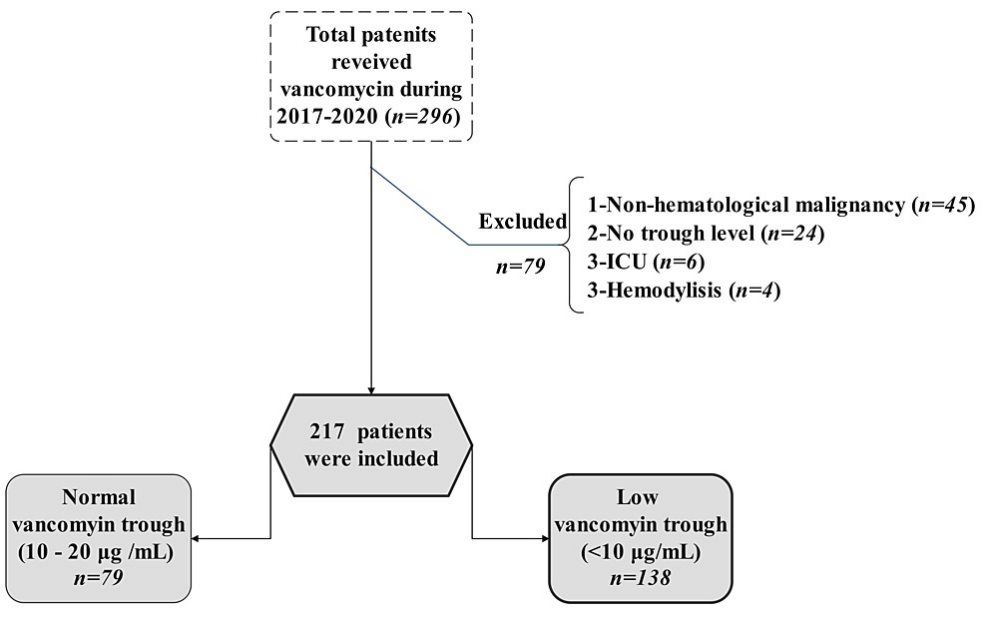
Flow chart of patient selection.

A total of 296 patients were screened: 217 (73.3%) were included and 79 (26.7%) did not meet the study criteria. The majority of the excluded patients were removed due to diagnosis with a non-HM, followed by missing clinical data (56.9% and 30.3%, respectively). Details regarding patient inclusion and exclusion are provided in Figure 1.

Table 1 represents the demographic data and clinical characteristics of the enrolled patients. The age and weight medians in the FN and non-FN patients were 42 years and 48 years as well as 67 kg and 71 kg, respectively. The patients who received vancomycin empirically or for a documented infection were divided into two groups: a febrile neutropenic group (n = 158, 72.80%) and a non-neutropenic group (n = 59, 27.20%). There were more patients diagnosed with leukemia than with lymphoma (64.50% vs. 28.60%), while 6.90% of the patients had multiple myeloma. The mean vancomycin dose and the corresponding mean trough in the neutropenic group were 30 mg/kg/day and 8.20 µg/mL, respectively, whereas in the non-neutropenic group they were 29 mg/kg/day and 11.12 µg/mL, respectively.

**Table 1:** Baseline demographics and clinical characteristics. FN, febrile neutropenia; TDD, total daily dose, ARC, augmented renal clearance.

Variable	FN (n = 158)	Non-FN (n = 59)	p-Value
Gender			
Male, n (%)	87 (55.10)	38 (64.40)	0.2
Female, n (%)	71 (44.90)	21 (35.60)	0.2
Age, years, median (Q1–Q3)	42 (29.70–52.25)	48 (33–56)	< 0.5
BMI, median (Q1–Q3)	26 (22.60–29.90)	27.30 (21–27.30)	0.65
Weight, kg, median (Q1–Q3)	67 (60.0–83.30)	71 (58.50–85.50)	0.54
CrCl, mL/min, median (Q1–Q3)	124.50 (89–154.20)	101 (79.20–131.80)	< 0.05
Trough concentration, μg/mL (mean ± SE)	8.2 (0.36)	11.12 (0.71)	< 0.05
TDD, mg/kg/day (mean ± SE)	30 (0.55)	29 (0.90)	0.12
ARC n (%)	75 (83.3)	15 (16.7)	
Albumin concentration, g/L (mean ± SE)	31.79 (0.38)	31.47 (0.74)	0.67
Indication of vancomycin			
Empirical therapy, n (%)	151 (95.60)	33 (55.90)	
Documented infection, n (%)	7 (4.40)	26 (44.10)	
Pneumonia, unspecified, n (%)	5 (15.20)		
Catheter-related infection, n (%)	6 (18.20)		
Skin/wound infection, n (%)	9 (27.30)		
Bone infection, n (%)	4 (12.20)		
Meningitis, n (%)	3 (9)		
Other, n (%)	6 (18.20)		
Malignancy type, n (%)			
Leukemia, n (%)	140 (64.50)		
Lymphoma, n (%)	62 (28.60)		
Multiple myeloma, n (%)	15 (6.90)		

Association of the type of HMs and FN with low vancomycin trough concentration

**Figure 2:**
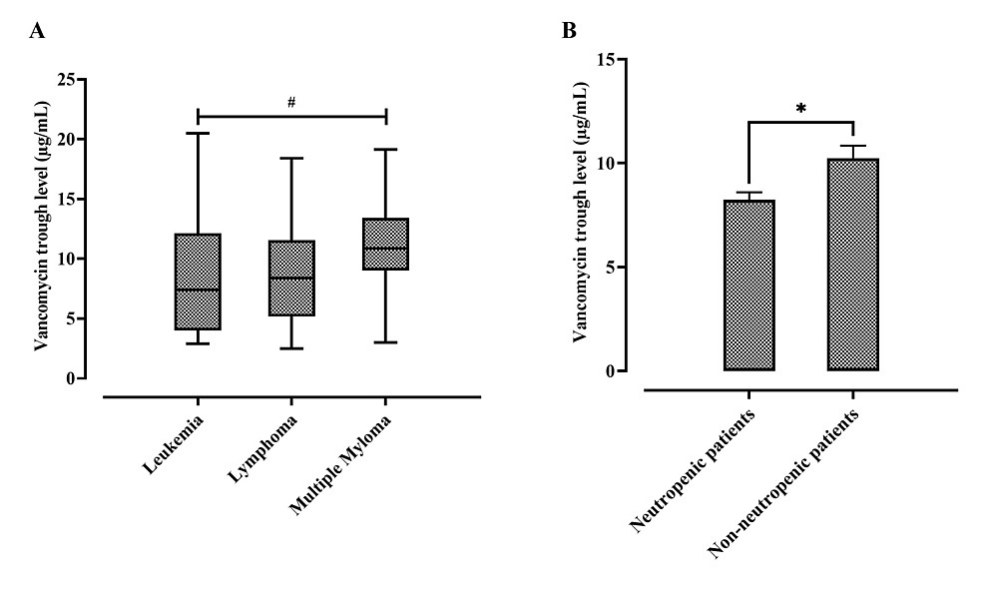
Difference in vancomycin trough concentration among (A) types of hematologic malignancies (5.5, 5.5, and 7.8 μg/mL) between leukemia, lymphoma, and multiple myeloma, respectively. # p > 0.05 between groups, using a Kruskal–Wallis test. Middle line: median; upper/lower box: upper or lower 25% of the data; and upper/lower bar: greatest or least value excluding outliers. (B) Neutropenic and non-neutropenic patients; * p < 0.05 compared with non-neutropenic patients' values, via a student’s t-test. Data expressed as mean ± SEM; n = 217.

A chi-square test of independence was performed to examine the association between different types of HM "leukemia, lymphoma, and multiple myeloma" and the incidence of low vancomycin trough concentration, and the result showed that there was no significant association: χ2 (2, N = 217) = 4.105, p > 0.05, with medians of 5.5, 5.5, and 7.8 μg/mL respectively (Figure 2A). However, a significant association between FN and the incidence of a low vancomycin trough concentration was observed: χ2 (1, n = 217) = 4.275, p < 0.05. Our results demonstrated an increase in the odds of low troughs among febrile neutropenic patients (OR = 1.89, 95% CI = 1.12 to 3.48, p < 0.05) as compared with non-neutropenic ones, with a mean ± SEM of 8.20 μg/mL ± 0.36 vs. 11.12 μg/mL ± 0.71; t (215) = -2.870, p < 0.05 (Figure 2B).

Possible underlying factors of a low vancomycin trough concentration among patients with FN

CrCl, BMI, age, gender, and albumin concentration were included in the logistic regression analysis for the factors associated with low trough concentrations among the FN patients; the predictors of a low vancomycin trough concentration were CrCl and a low albumin level, with adjusted odds ratios of OR = 1.04 (95% CI 1.022-1.061, p < 0.05) and OR = 1.11 (95% CI 1.040-1.215, p < 0.05), respectively.

Effect of ARC on vancomycin trough concentrations among patients with FN

**Figure 3:**
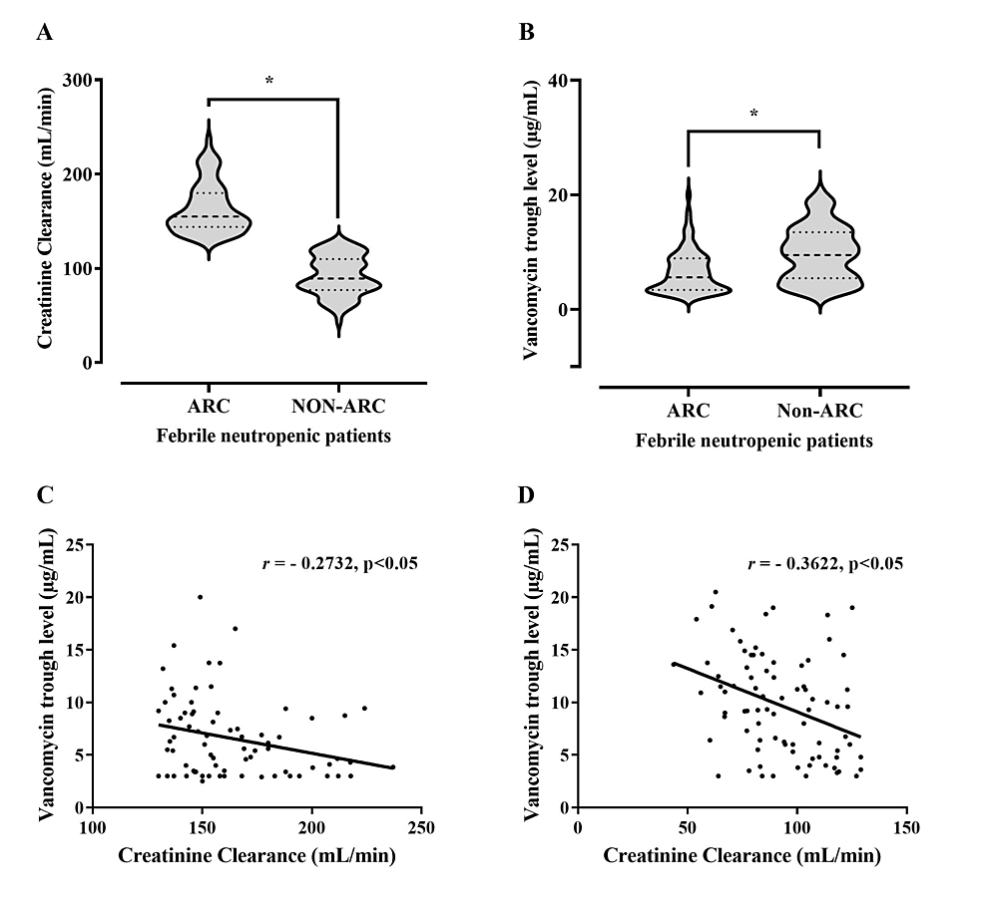
Difference between augmented renal clearance (ARC) and non-ARC groups. Difference between ARC and non-ARC groups in (A) creatinine clearance (mL/min) and (B) vancomycin trough concentration (μg/mL); * p < 0.05 compared with non-ARC values, via a Mann–Whitney U test. The center line of the violin represents the group median, while the upper and lower lines inside the violin represent the 75th and 25th percentiles, respectively. The correlation between vancomycin trough concentration and creatinine clearance was a statistically significant inverse correlation among febrile neutropenia (FN) patients with (C) ARC (r = –0.2732, p < .05) and (D) without ARC (r = –0.3622, p < .05).

The enrolled patients with FN were divided into two groups according to their CrCl: patients with ARC, n = 75 (47.50%), and without ARC, n = 83 (52.50%). The χ2 has shown that there is a significant association between FN and ARC: χ2 (1, n = 217) = 8.60, p < 0.05. In addition, the proportion of the coexistence of FN with ARC was 83.3%. The CrCl for FN patients with ARC was significantly higher (p < 0.05) than for those without ARC, with a median of 155 mL/min vs. 89.30 mL/min (Figure 3A). Furthermore, the trough concentration among FN patients with ARC was significantly lower (p < 0.05) than for those without ARC, with a median of 5.60 μg/mL vs. 9.64 μg/mL (Figure 3B), as were the odds of low troughs among FN patients with ARC (OR = 5.5, 95% CI = 2.52 to 11.48, p < 0.05) as compared to FN patients without ARC. There was a statistically significant inverse relationship between trough concentration and CrCI among FN patients with or without ARC (r = -0.2732, p < .05 vs. r = -0.3622, p < .05) (Figures 3C, 3D).

Moreover, the logistic regression analysis identified the risk factors associated with ARC, including the male gender, FN, age ≤ 30, and BMI > 30 (Table 2).

**Table 2:** Risk factors associated with ARC. FN, febrile neutropenia; BMI, body mass index, ARC: augmented renal clearance

Independent Risk Factors	Adjusted OR	CI 95%	p-Value
Gender (male)	3.4	1.7–6.7	0.000
FN	3.46	1.5–7.8	0.003
Age (≤ 30)	10.15	4.4–23	0.000
BMI (> 30)	2.9	1.35–6.3	0.006

## Discussion

In this study, we evaluated the association of vancomycin's low trough concentration in patients with an HM in the presence or absence of FN. Our findings showed that the mean trough concentration of vancomycin-treated patients with FN was 8.20 µg/mL, whereas in the non-neutropenic group it was 11.12 µg/mL. Furthermore, FN was associated with low vancomycin concentrations. Interestingly, ARC was observed in the majority of the FN patients with a significant association, which might be considered an additional risk factor for a low trough in neutropenic patients.

Our findings revealed there was no significant association between the type of hematological malignancy and the incidence of a low trough concentration (p > 0.05), however, there was a significant association between FN and the incidence of a low vancomycin trough concentration (OR, 1.89, p < 0.05). This result is in agreement with Haeseker et al., who reported neutropenia as a primary independent predictor of enhanced clearance. Five patients who received two courses of vancomycin during a neutropenic and non-neutropenic phase were included in the subgroup analysis. According to the results, vancomycin clearance was significantly greater during the neutropenic phase as compared to the non-neutropenic phase (91 ml/min vs. 45 ml/min, p = 0.009). This finding highlighted the need to increase the daily dose of vancomycin by 30% in patients with FN, from 15 mg/kg twice daily to 13 mg/kg three times per day. In line with this, Choi et al. found an association between neutropenia and subtherapeutic vancomycin concentrations [19,20]. Similarly, Zahrani et al. demonstrated that febrile neutropenic patients are 2.4 times more likely to have a low trough concentration than non-neutropenic patients [21]. Additionally, Hochart et al. conducted a retrospective study on 54 patients with acute myeloid leukemia (AML) who received 67 sessions of vancomycin. The primary objective of this research was to determine the percentage of patients who had an optimal vancomycin concentration at the time of the first or subsequent monitoring of their blood concentration. They reported that the target concentration was only attained in 32% of cases after a mean 1.5 dose adjustment, despite the higher dose. The mean final maintenance dose was 42.1 ± 9.4 mg/kg per day [2]. In addition, several descriptive investigations found that the standard vancomycin dose in neutropenic patients was insufficient for optimal antibacterial efficacy. Despite the development of various pharmacokinetic models and the proposal of a few dosage regimens, there is no consensus on the initial vancomycin dose in patients with hematologic malignancies or neutropenia. None of the international guidelines provide specific recommendations for this subpopulation [4,22].

Our present analysis found that CrCl and a low albumin concentration were associated with a low trough among the FN patients (OR of 1.04 and 1.11, respectively). These findings are consistent with previous work by Choi et al., 2017, which revealed that neutropenia (OR, 1.75), BMI (OR, 1.13), and hypoalbuminemia (OR, 1.13) were associated with low troughs by using logistic regression analysis [23]. However, these results might shed light on the idea that low trough concentrations in febrile neutropenic patients are attributed to a multifactorial mechanism rather than one factor.

The association of hypoalbuminemia and a low vancomycin trough concentration might be explained by the higher ratio of free/plasma-protein-bound fractions of vancomycin, “protein binding: ~55%” [24], which in turn leads to the movement of the free molecules outside the circulation, distributed more into the tissues, and resulted in an increased volume of distribution. Generally, increases in the volume of distribution are likely to gradually decrease the vancomycin maximum (Cmax) and total concentrations. During the elimination phase, the kidneys can only eliminate free molecules. It seems likely that rising unbound vancomycin molecules appear to result in a boosted total renal clearance. A low vancomycin concentration is the result of not only the fact that FN patients are more likely to have a higher CrCl than non-neutropenic patients but also a higher unbound fraction of vancomycin due to low albumin, resulting in augmented vancomycin clearance and lower total concentrations during the elimination phase [25-27]. Other factors that have been reported to have an association with vancomycin binding need to be considered, such as bilirubin [28].

Our findings illustrated a higher incidence of ARC: 83.3% in FN patients. Additionally, our results demonstrated the odds of low troughs among FN patients with ARC (OR = 5.5, 95% CI = 2.52 to 11.48, p < 0.05) as compared to FN patients without ARC.

ARC has been described in conditions of sepsis, trauma, burns, traumatic brain injury, subarachnoid hemorrhage, and in hematological malignancy patients with febrile neutropenia. Furthermore, the incidence of ARC in critically ill patients has been reported in previous studies to be from 16% to 41% [12].

While most previous studies determined the risk factors for ARC among critically ill patients, burn patients, and neurotrauma patients, the current study determined the risk factors for ARC among HMs without mixed conditions [9,12,13]. To our knowledge, no such analysis has been conducted in this group of patients. Our work demonstrated that holding all other predictor variables constant, the odds of ARC increased by 240%, 245%, 915%, and 190% for the male gender, FN, age ≤ 30, and BMI > 30, respectively. In other words, febrile neutropenia is an independent risk factor for augmented renal clearance in our patients. These findings suggested that not all patients were at the same risk for ARC, and highlighted the need to screen for ARC risk factors in specific patient populations. Udy et al. recently showed that age < 50 years, a diagnosis of trauma, and a modified Sequential Organ Failure Assessment (SOFA) score are the most important risk factors in ICU patients [13]. Similarly, Hefny et al. reported that age, the male sex, and trauma were significantly associated with ARC in critically ill patients [29]. Additionally, Minkute et al. reported that younger age, in addition to mechanical ventilation and the hemodynamical status of a patient, were significant risk factors. In contrast to earlier findings, researchers could not confirm that ARC occurred more often in males [30]. Moreover, Zhao et al. identified risk factors associated with ARC in Chinese adult patients, which included the male gender, age < 50 years, overweight, receiving mechanical ventilation, enteral nutrition, neutrophil percentage, and cardiovascular diseases [31].

The exact mechanism by which FN might induce ARC is not yet fully understood; however, one theory suggested that the development of a systemic inflammatory response syndrome (SIRS) may be responsible for this association. A SIRS is associated with the production of inflammatory mediators, which may significantly enhance cardiac output and lower vascular resistance, increasing renal blood flow and, therefore, glomerular filtration rate (GFR). This may be increased further by the use of high-volume fluid therapy, which is common in patients diagnosed with cancer, particularly for patients who need post-chemotherapy hydration [13].

Finally, some important limitations need to be considered in the current work. First, using the Cockcroft-Gault equation to calculate CrCl has been shown to completely underestimate the real CrCl compared with a matching estimation by using an eight- to 24-hour urine sample measurement [29]. Second, since the study was conducted retrospectively at a single tertiary care facility, it is difficult to extrapolate the findings to a general population. Third, the initial steady-state concentration of vancomycin troughs has been analyzed in this study after empirical or therapeutic treatment. This study did not evaluate patient outcomes, such as the cure rate. Finally, data were collected via the computerized hospital system; therefore, no interventions were performed on the enrolled patients. Such investigations only enable the identification of the association between study variables, so these results, therefore, should be interpreted with caution.

## Conclusions

The findings of this study conclude that FN is associated with low vancomycin concentrations. Interestingly, ARC was observed in most patients with FN with a significant association, which may be considered the main driver for a low trough in neutropenic patients. It is recommended that further research be undertaken to assess the effects of neutropenia on each pharmacokinetic parameter (Vd, Cl, Ke, etc.). This will easily facilitate the interpretation of kinetics-guided dose calculations for such patients.
